# Gut Dysbiosis with Minimal Enteritis Induced by High Temperature and Humidity

**DOI:** 10.1038/s41598-019-55337-x

**Published:** 2019-12-10

**Authors:** Song Chen, Yuhua Zheng, Yiqing Zhou, Weizhong Guo, Qin Tang, Guangli Rong, Weiwei Hu, Jianbang Tang, Huanhuan Luo

**Affiliations:** 1 Institute of Tropical Medicine, Guangzhou University of Chinese Medicine, No.12 Jichang road, Baiyun district, Guangzhou City, Guangdong Province People’s Republic of China; 2 Zhongshan Hospital of Chinese Medicine, No.3 Kangxin road, Xi district, Zhongshan City, Guangdong Province People’s Republic of China; 3 School of Basic Medicine, Guangzhou University of Chinese Medicine, No. 232 Wai Huan Dong Road, University Town, Panyu District, Guangzhou City, Guangdong Province People’s Republic of China

**Keywords:** Inflammation, Chronic inflammation

## Abstract

High temperature and humidity (HTH) can cause diarrhea owing to food and drinking water contamination. However, their direct effects on gut microbiota and gastrointestinal inflammation are unknown. This study aimed to investigate the effects of HTH and probiotics on the microbiome. Twenty-one male mice were randomly assigned to normal control (NC), HTH, and broad-spectrum probiotic-treated (PR) groups. HTH and PR groups were regularly housed at 30 ± 0.5 °C with humidity of 85–90% for eight consecutive weeks. A broad-spectrum probiotic was administrated to PR-group mice from day 50 to 56. Clinical signs were observed and gut microbiota were analyzed via 16 S rRNA-based functional metagenomics. Intestinal pathology and the expression of defensins and pro-inflammatory cytokines were also assessed. Mice in the HTH and PR groups gradually developed sticky or loose feces. The HTH group developed a distinct microbiota profile associated with augmented metabolism and human-like pathophysiologies upon suppression of environmental sensing. Pathological assays indicated minimal enteritis, increased bacterial translocation, and elevated intestinal pro-inflammatory cytokine levels. Thus, ambient HTH directly contributes to gut dysbiosis and minimal enteritis, whereas probiotics partially normalized the microbiota and ameliorated gut inflammation. This study provides novel insights into the pathogenesis of environment-associated diseases and offers a potential therapeutic approach.

## Introduction

The effect of climate on health has received increasing attention. Diarrheal disease is the leading cause of childhood morbidity and mortality worldwide, with an estimated 1.7 billion infections and 0.7 million deaths occurring annually^1^. Previous studies have reported that climate-related factors significantly affect seasonal diarrhea in susceptible populations. A two-fold increase in daily hospital admissions for diarrhea has been observed for every 5 °C increase in the mean ambient temperature^2^. Higher temperatures reportedly increase bacteria- and parasite-induced diarrhea and contribute to the survival of enterogastritis-causing bacteria such as *Escherichia coli* in contaminated food^3^. Similarly, relative humidity significantly contributes to diarrhea-associated morbidity, probably owing to the compromised efficiency of drinking water treatment plants and contaminated water distribution systems during heavy rain^4,5^. However, whether a high ambient temperature and humidity (HTH) directly affect mucosal immunity and the gut microbiota, thus causing diseases including diarrhea, are unclear.

The Ling’nan region of South China is an ideal region to study the effect of climate on health. This region encompasses the southern region of the Nanling Mountains and covers the Guangdong, Guangxi, and Hainan provinces, representing the hottest and most humid area with the most ideal conditions for diarrhea (peak time) among 31 provinces in China^6^. As predicted by Chinese medicine, people living here often suffer abdominal discomfort and mild diarrhea in early summer. Most interestingly, most of them denied the intake of contaminated food or water. Although the underlying pathomechanism remains unknown, according to Chinese medicine, extreme relative humidity potentially approaching 100% and lasting a month directly causes discomfort.

This study aimed to investigate the effects of HTH and probiotics on the microbiome in 21 male mice randomly assigned to normal control (NC), HTH, and a broad-spectrum probiotic-treated (PR) groups. Our results may provide novel insights into the pathogenesis of climate-associated diseases, for which the gut microbiota could be considered a promising therapeutic target.

## Results

### Clinical manifestations and histological changes in the colon of mice

**Figure 1:**
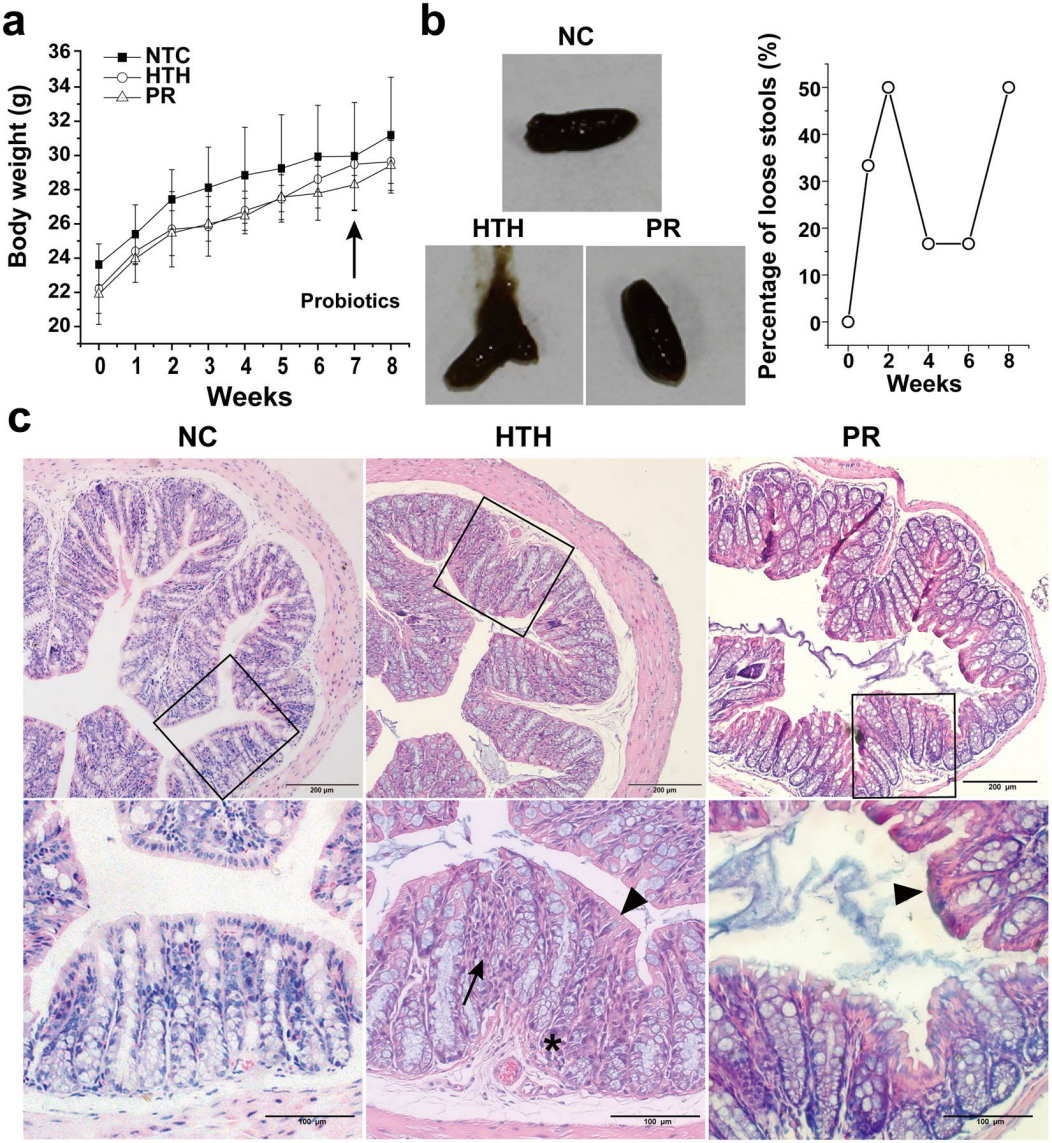
Body weight, appearance of faeces, and histological changes in the colon. (**a**) The curve of the increase in body weight (*Mean* ± *S.D*). (**b**) Appearance of loose faeces in the HTH group compared to the NC group. (**c**) Histological changes in the colon in the NC group and HTH groups. A destroyed crypt (asterisk), infiltrated with inflammatory cells in the lamina propria (long arrow), and thinning of the epithelial layer (arrow head). H&E. X200.

Throughout the experiments, the body weights of all animals increased gradually with no differences among groups (Fig. 1a). Control mice were very active, had sleek coats, and excreted solid feces. In contrast, mice maintained in the climate chamber (HTH and PR groups) gradually exhibited reluctance to move, reduced feeding, unkempt and dull coat, and sagging scrotum. After 1 week of probiotic treatment, these signs significantly improved in the PR group. Interestingly, most mice transferred into the climate chamber developed sticky stool, and approximately half of them secreted loose feces (Fig. 1b), which is defined as pasty and semi-formed stools that do not stick to the anus as Copper *et al*. has described^7^. In the HTH group, loose stools peaked in the first 2 weeks, gradually subsided, then rebounded after 6–8 weeks (Fig. 1b). No loose stool was observed in the NC and PR groups after 8 weeks. Because diarrhea was rare, no gross bleeding was observed, the hemoccult positivity test was not performed, and the disease activity index was not calculated as described by Copper *et al*.^7^. No death occurred during our experiments.

Furthermore, we compared the histological changes in the colon between the NC and HTH groups. Using light microscopy, the histologic changes observed in the colon of the HTP group included destroyed crypts (asterisk), inflammatory infiltrate in the lamina propria of the mucosa (long arrow), and thinning and disorganization of the epithelial layer (arrowhead) (Fig. 1c). These histological changes in the colon were partially alleviated in the PR group. However, shortening of the crypts, the most typical feature in dextran sulfate sodium-induced colitis and that acts as a critical parameter for lesion grading, was not observed in our study. We therefore did not compare the lesion grade of colitis as described^7^.

### Microbiota profiles

**Figure 2:**
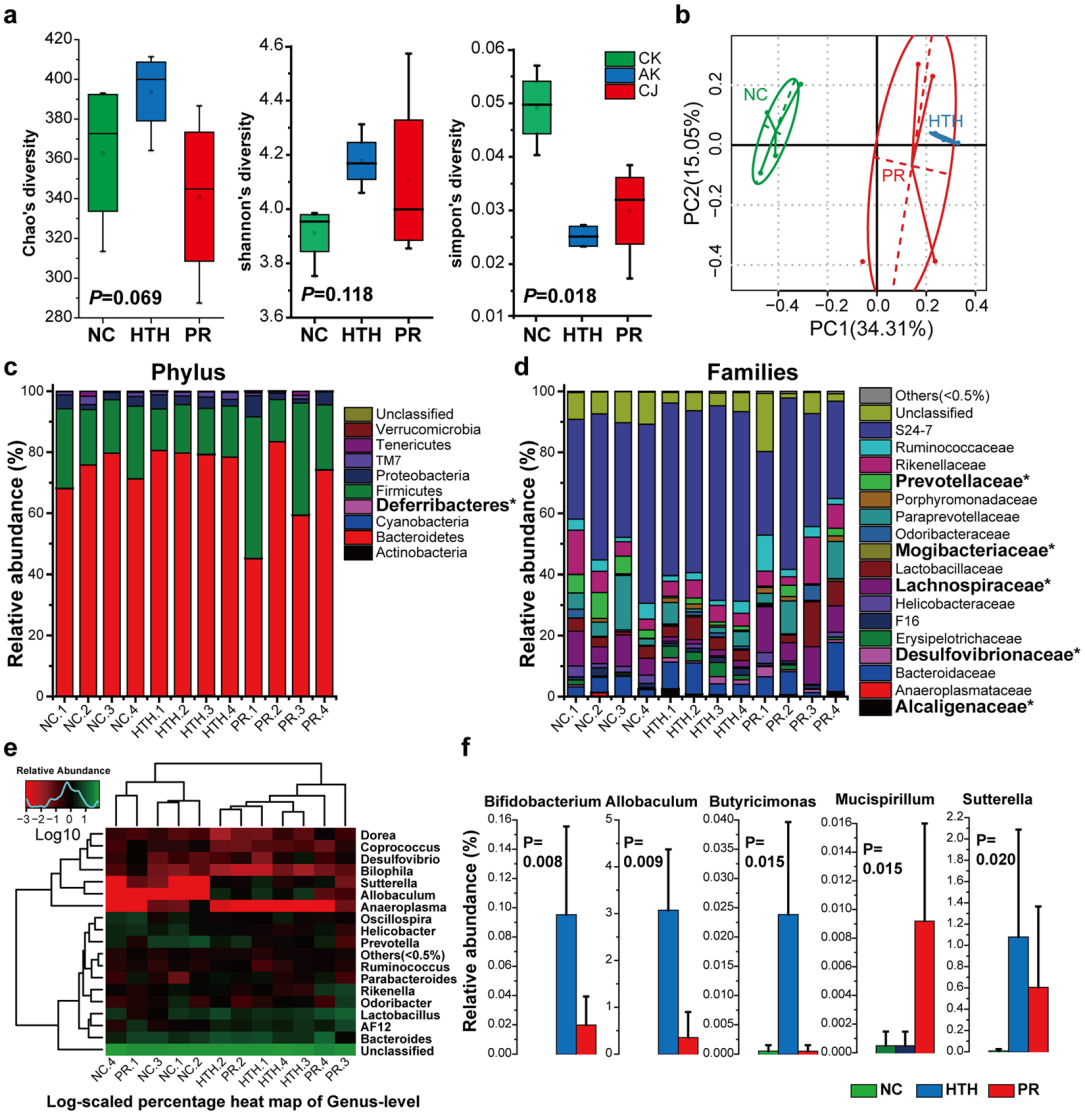
Community distribution of the faecal microbiota based on 16 s rRNA gene surveys. (**a**) Alpha diversity was applied to analyse the complexity of species of the NC, HTH, and PR groups. Indices of Chao1, Shannon, and Simpson are displayed. (**b**) Principal component analysis (PCA) at the Operational Taxonomic Units (OUT) level. The PCA of all the samples was based on the relative abundance of the OTUs. Numbers in brackets represent the contributions of principal components to differences among samples. (**c**) The abundance of the different phyla, and (**d**) different families in all samples was shown. “*”: Phyla and families with different abundance are highlighted in black. (**e**) A heat map showing the taxonomic composition distribution among samples at the genus-level. (**f**) Five bacterial genera are identified with significant different abundance among groups.

A total of 1,491,686 high-quality reads with an average length of 252 bp was obtained from 12 fecal samples. Those reads were combined into 742,961 tags with 61,913 tags per sample on average. To assess overall differences in microbial community structure among the groups, we measured ecological parameters based on alpha-diversity (ChaoI, Shannon, and Simpson indices). As shown in Fig. 1a, the mean values of both the ChaoI index and Shannon index increased in the HTH group, whereas these values decreased after probiotic treatment (PR group). Consistently, the mean value of the Simpson’s index declined significantly (*P* < 0.05) in the HTH group, but was partially restored in the PR group. To display differences in OTU composition among different samples, PCA was used to construct a 2-D graph summarizing factors primarily responsible for this difference. Weighted UniFrac analysis revealed that the first coordinate (PC1) elucidated 34.31% of the inter-sample variance (*P* = 0.01; Fig. 2a), whereas unweighted UniFrac analysis elucidated 15.05% of differences between the HTH and NC groups (*P* = 0.01; Fig. 1b).

When grouping OTUs at the phylum level, we considered the relative abundances of the nine phyla (e.g., Actinobacteria, Bacteroidetes, Cyanobacteria, Deferribacteres, Firmicutes, Proteobacteria, TM7, Tenericutes, and Verrucomicrobia). In all three groups, Bacteroidetes and Firmicutes were the most abundant bacterial phyla (Fig. 2c). Relative abundances of these phyla were compared among the three groups using the Kruskal-Wallis test, and Deferribacteres was identified as the only phylum with differential abundance (*P* < 0.05), increasing from 0.05% in the NC and HTH groups to 1% in the PR group. The ratio of Bacteroidetes/Firmicutes also tended to increase in the HTH group and decrease in the PR group, although the differences were not significant.

At the family level, three families (Alcaligenaceae, Desulfovibrionaceae, and Mogibacteriaceae) increased, whereas two families (Lachnospiraceae and Prevotellaceae) decreased in the HTH group compared to those in the NC group upon comparison of OTUs. At the genus level, 11 bacteria were identified with significantly different abundances (*P* < 0.01) among the three groups. The top five significantly different genera among the three groups were *Bifidobacterium, Allobaculum, Butyricimonas, Mucispirillum, and Sutterella* (Fig. 2e,f). Although no significant differences in taxa were described among the three groups with an FDR correction of *P*-values (FDR = 0.116 for all the five genera),these results implied candidate panels to explain dysbiosis in the HTH group. Although *Mucispirillum* was not present in the probiotic product administrated in our study, it was induced significantly upon probiotic treatment, suggesting that the probiotics probably exert their beneficial effects via a mechanism independent of their intestinal colonization.

### Functional prediction using the KEGG ortholog database

**Figure 3:**
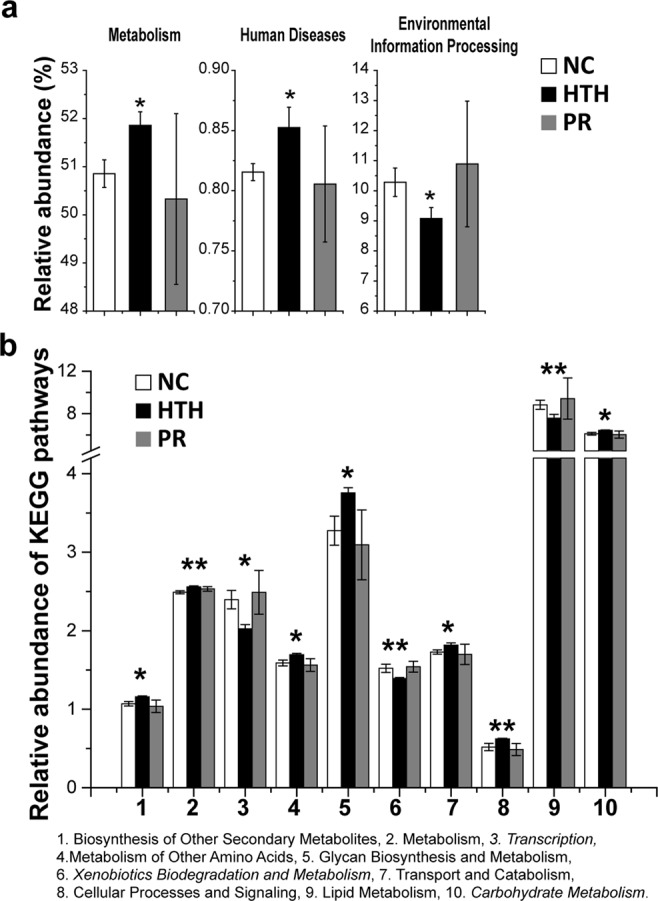
Functional analysis of the gut microbiota using PICRUSt based on the 16 S rRNA data. (**a**) Columns show 3 differential abundant categories of KEGG pathways. (*P < 0.05) (**b**) The subdivision of KEGG pathways with differential relative abundance are shown in a bar plot. (*P < 0.05).

Based on PICRUSt, changes in the functional capacity of the gut microbiota, as indicated through KEGG pathways, were predicted. Metabolism and human disease pathways were enriched and the environmental information processing pathway was suppressed, in the HTH group compared to those in the NC group; this trend was partially reversed upon probiotic treatment (Fig. 3a). At KEGG level 2, 10 predicted pathways in total were significantly differentially regulated. These pathways were mostly associated with augmented metabolism and biosynthesis (pathways 1, 2, 4, 5, 7, 8, and 10 in Fig. 3b), and diminished transcription, xenobiotic biodegradation and metabolism, and lipid metabolism (pathways 3, 6, and 9 in Fig. 3b).

### LPS translocation and pathological manifestations of minor enteritis in the gut

**Figure 4:**
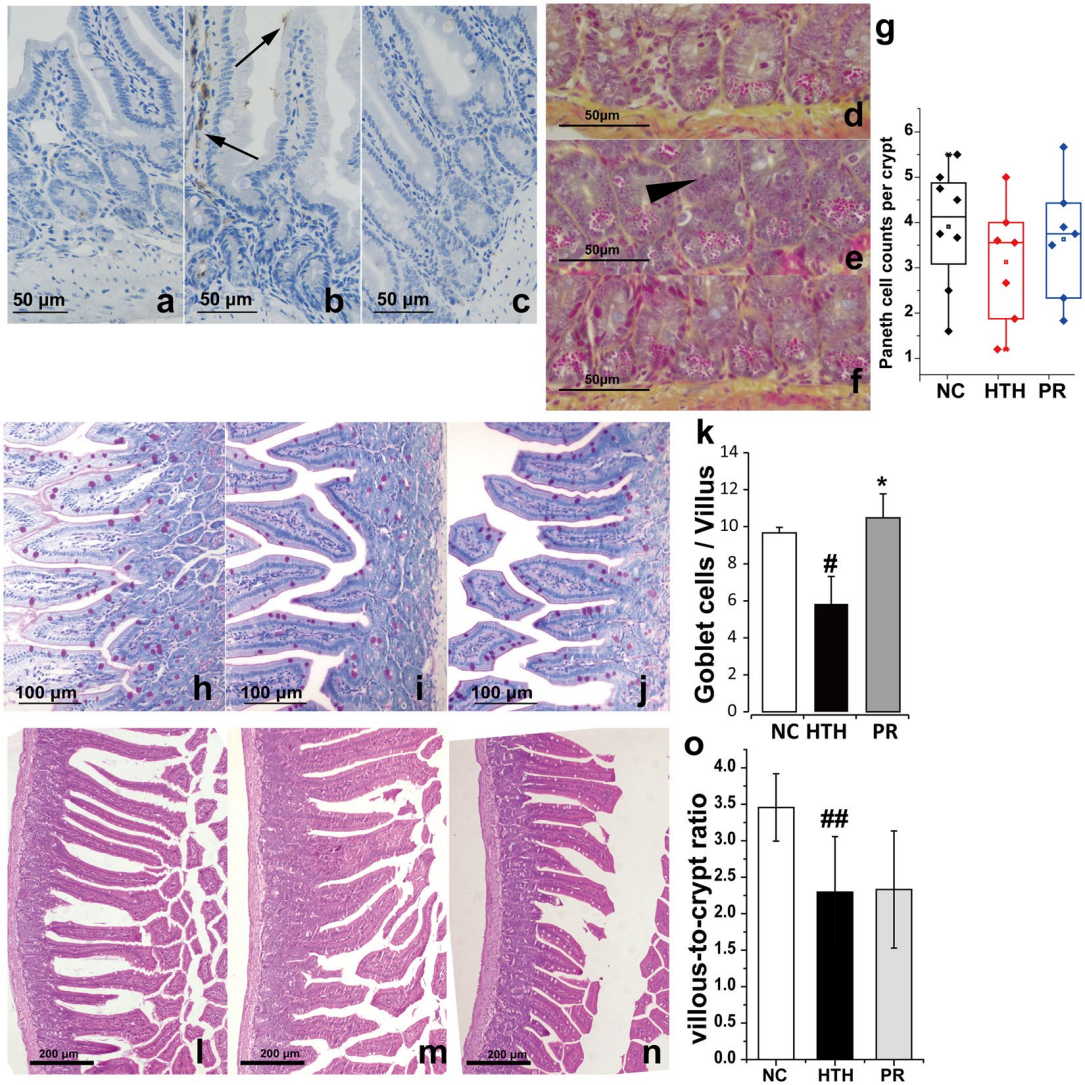
Histomorphology of ileal tissue in mice. (**a–c**) Immunohistochemical staining of LPS in ileal tissue of mice in the NC (**a**), HTH (**b**), and PR (**c**) groups. Long arrows indicate LPS-positive sites. DAB staining and counterstaining with hematoxylin. (**d–f**) Phloxine-tartrazine staining of Paneth cells in the NC (**d**), HTH (**e**), and PR (**f**) groups. Arrow head indicates diffuse or excluded granules. (**g**) Counts of phloxine-tartrazine stained Paneth cells per crypt are shown. (**h–j**) PAS staining for (**h**) NC, HTH (**i**), and PR (**j**) groups. (**k**) Counts of PAS-positive cells are shown in columns (*Mean* ± *S.D*.). (**l–n**) Representative histomorphology of the intestine in the NC (**l**), HTH (**m**), and PR (**n**) groups (H&E). (**o**) Villous-to-crypt length ratios are shown in columns (*Mean* ± *S.D*.). ^#^, ^##^: compared to NC group, *P* < 0.05, *P* < 0.01, respectively; *: compared to HTH group, *P* < 0.05.

As the excretion of sticky feces was one of the most prominent symptoms of HTH treated mice, suggesting abnormal lipid metabolism, and KO analysis also predicted impaired lipid metabolism, we assessed the events occurring in the small intestine upon HTH. Immunohistochemical staining of LPS was performed to elucidate bacterial translocation. At 56 days, a remarkable increase in LPS adhering on the villar epithelium or penetration into the villa and lumen were observed in the HTH group (long arrow), compared to the NC group, whereas LPS staining tended to be reduced after probiotic treatment (PR group; Fig. 4a–c).

We further investigated the changes in the Paneth and goblet cells to ambient HTH. As reported previously, the response of Paneth cells to pathological conditions may vary^8^. Cell numbers can decrease owing to non-specific injury during inflammatory conditions or increase in regions undergoing regeneration and repair, whereas cytoplasmic lysozyme staining might be normal, disordered, depleted, and diffuse^8,9^. In our study, compared with the NC group, the count of phloxine-tartrazine-positive Paneth cells per crypt tended to decrease (Fig. 4g, *P* > 0.05) in mice of the HTH group accompanied with diffuse or excluded granular phenotypes (arrow head). The diffused granules seemed to decrease after probiotics treatment (PR group) (Fig. 4d–f). Goblet cells were also apparently reduced in number and size (Fig. 4h–j).The Goblet cell count per villus in the HTH group decreased significantly compared to the NC group, whereas it recovered in the PR group (*P* < 0.05) (Fig. 4k).

Based on H&E staining and pathological examination (Fig. 4l–n), a mild “villous blunting” (defined by villous-to-crypt length ratio of 2:1 to 3:1^10^) could be observed in the HTH and PR groups (*P* < 0.01, Fig. 4o). The villus length was measured from the villus tip to the villus-crypt junction, whereas crypt depth was defined as the depth of the invagination between two villi. “Villous blunting” is a commonly used criterion for evaluating pathological changes in the intestinal inflammation^10^. T confirm these results, we repeated the experiments in which animals were assigned into the NC and HTH groups, and treated similarly. Compared to the NC group, the villous-to-crypt length ratio in the HTH group also decreased significantly (*P* < 0.01), as shown in Supplementary Fig. 1. Moreover, based on H&E staining and pathological examination, the minimal intestinal inflammation signs including the loss of epithelium and the inflammatory cells infiltration into the lamina propria could be observed in the HTH group (Supplementary Fig. 2)

### Intestinal expression of defensins, pro-inflammatory cytokines, and systemic inflammation

**Figure 5:**
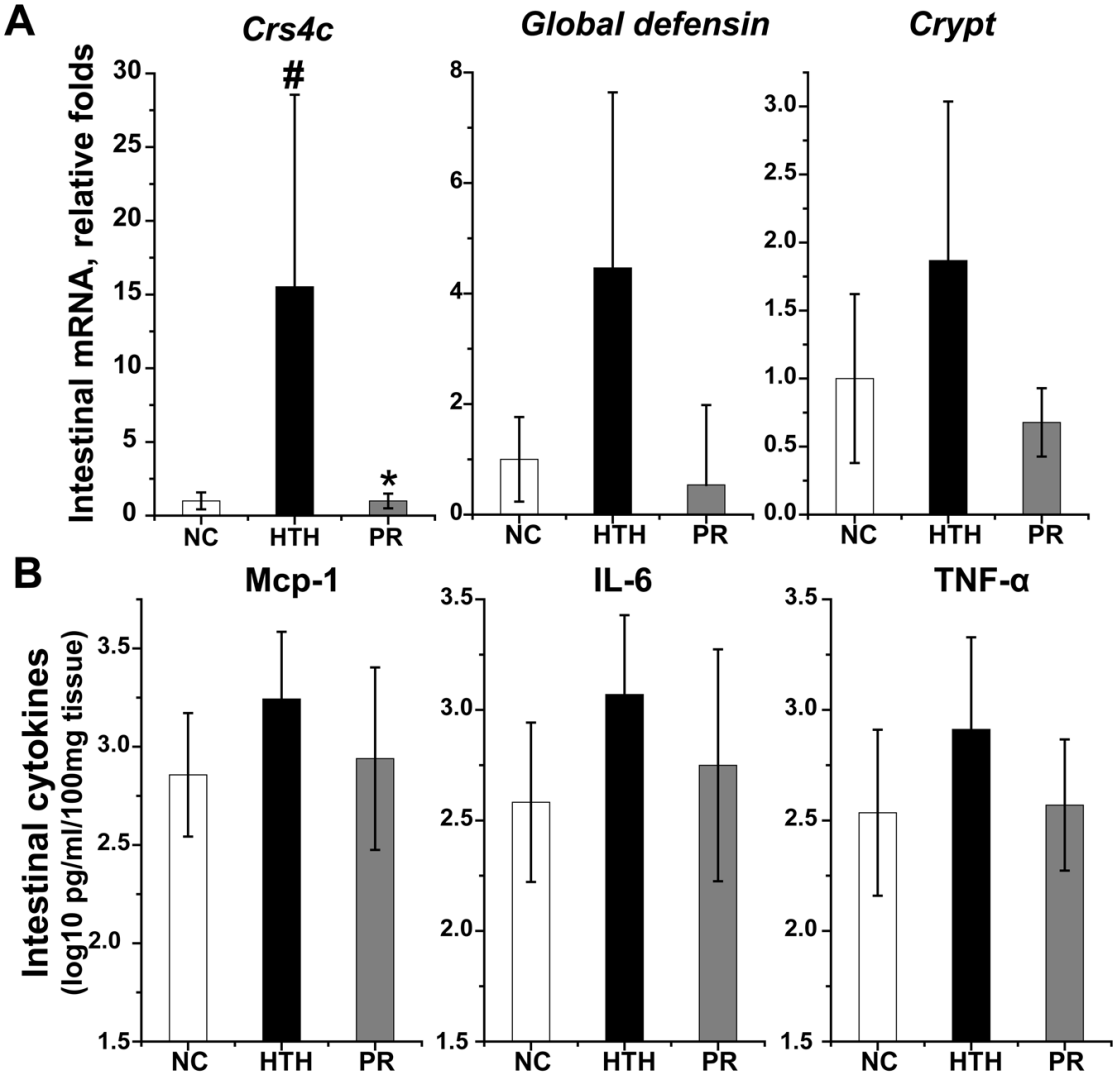
Expression levels of intestinal defensins and pro-inflammatory cytokines. (**A**) Expressions of intestinal defensins (including *CRS4C*, *global defensin*, and *cryptin1*) are shown. (*Geomean* ± *S.E.M)*. (**B**) MCP-1, TNF-α, and IL-6 in the supernatant of intestinal homogenates were quantitated using an ELISA assay. (*Mean* ± *S.D*, normalized to 100 mg intestinal tissue). ^#^: compared to NC group, *P* < 0.05; *: compared to HTH group, *P* < 0.05. NC: normal control group, HTH: high temperature and humidity group, PR: probiotics group.

Considering the abnormal histological appearance of Paneth cells, we determined whether the impaired function of these cells resulted in the impaired secretion of defensins, contributing to LPS translocation. Intestinal mRNA levels of *CRS4C* were significantly higher in the HTH group than in the NC group (*P* < 0.05), suggesting inflammation-associated activation instead of impaired Paneth cell function (Fig. 5A). Similarly, intestinal expression of global defensins and cryptin 1 tended to increase in the HTH group; all three defensins tended to decline upon probiotic treatment, although the difference was not significant.

Local inflammation induced by increased bacterial translocation potentially contributes to the upregulated expression of defensins in Paneth cells. As an agonist of TLR2 and TLR4, LPS serves as a potent stimulator of NF-κB signaling and induces the expression of pro-inflammatory cytokines, including MCP1, IL-6, and TNF-α. Using intestinal tissue homogenates, the levels of these three cytokines tended to increase in the HTH group compared to those in the NC group and were restored upon probiotic treatment, although the difference was not statistically significant (Fig. 5B).

## Discussion

Factors related to climate significantly affect seasonal diarrhea, which is commonly considered to result from increased contamination of food and water. Nevertheless, the direct effect of temperature and humidity on the human body and its relevance to diarrhea was previously unclear. In this study, using a climate chamber, we mimicked a high temperature/humidity environment for 12 h per day for mice, and observed the gradual development of sticky and loose feces, even though careful measures were taken to prevent bacterial contamination in food and water. In addition, we observed a distinct pattern of gut dysbiosis, increased bacterial translocation, minimal enteritis, and elevated systemic levels of the pro-inflammatory cytokine TNF-α. Our results may explain the pathogenesis of some episodes of patient discomfort, especially slight fever and diarrhea, which commonly occur in the summer. To our knowledge, this is the first description of the induction of gut dysbiosis with climate-related factors. Consistent with this hypothesis, our results elucidate the beneficial effects of probiotics on dysbiosis and intestinal inflammation, which suggests a novel strategy to treat climate-associated discomfort by modifying the gut microbiota.

Furthermore, we attempted to characterize HTH-induced dysbiosis. Unexpectedly, we revealed a distinct pattern of increased gut microbiota diversity, indicated by increased ChaoI and Shannon indices and a decreased Simpson index in the HTH group. Increased diversity is associated with protective effects against some human diseases^11,12^. Consistently, KEGG analysis indicated an increase in metabolism in our HTH group. Paradoxical results between increased microfloral diversity and clinical symptoms could be explained on the basis of the overlapped effect of high temperature and humidity. In another preliminary experiment, high humidity caused remarkable loss of microbial diversity whereas appropriate high temperature reversed it (data not shown). Although the optimal housing temperatures for mice to mimic the thermal environment of humans are still debated, laboratory mice under standard conditions (21 °C) are under constant cold stress^13^. As reported previously, 30 °C^14^, the thermoneutral zone of mice, has been suggested, whereas other studies indicate that 30 °C is too warm^15^. Hence, we selected 30 °C as our experimental setting as it is a relatively “high temperature,” especially considering the tolerance of mice under high humidity. Finally, our results suggest that an adequate increase in ambient temperature to approximately 30 °C is potentially beneficial for gut microfloral diversity.

Taxonomically, families Alcaligenaceae, Bifidobacteriaceae, Desulfovibrionaceae, and Mogibacteriaceae were enriched and Lachnospiraceae and Prevotellaceae were reduced. At the genus level, we identified 11 bacterial genera with significantly different abundances among the groups, among which the top five enriched genera were *Allobaculum*, *Bifidobacterium*, *Butyricimonas*, *Mucispirillum*, and *Sutterella*. Unexpectedly, most of those genera are considered to protect against insulin resistance^16^, diabetes, and colitis^17^. Many studies have indicated that butyrate-producing bacteria are essential for maintaining gut integrity^18,19^ and have revealed significant negative correlations between the abundance of *Butyricimonas* and *Bifidobacterium* and plasma glucose and insulin levels^20^. Some studies have reported the detrimental effect of *Allobaculum* and *Sutterella* on the GI tract. *Allobaculum*^21^ was reportedly enriched in a Gulf War Illness (GWI)-model, a model displaying neurological abnormalities, neuroinflammation, chronic fatigue, and gastrointestinal disturbances, which are reportedly associated with Gulf War chemical exposure. In this model, an altered microbiome significantly decreased the tight junction protein occludin with a concomitant increase in claudin-2, characteristic of a leaky gut. High levels of intestinal, mucoepithelial-associated *Sutterella* species are also considered to be associated with autism, which is usually accompanied by GI disturbances^22,23^.

The effects of climate-related factors on glucose metabolism have been observed for many years. Mice exposed to a persistent environment of high temperature and humidity were found to develop hypoglycemia^24,25^ via unknown mechanisms. In this study, the enriched gut microbiota was predicted using KO analysis and was functionally associated with multiple metabolism-related pathways, thus potentially accounting for hypoglycemia induced by high temperature and humidity. Most importantly, the metagenome profile predicted diminished membrane transport and transcriptional pathways, which could have contributed to the development of enteritis in the HTH group. Thus, our study provides novel insights into the pathogenesis of climate-associated diseases, and suggests that the gut microbiota is a potential therapeutic target in these conditions.

## Materials and Methods

### Ethical approval

#### Animals

C57Bl/6 mice (6-weeks-old) were obtained from and maintained in the Laboratory Animal Research Center of Guangzhou University of Chinese Medicine (GZUCM, Guangzhou, China). Male mice were used in this study to minimize potential confounding effects of the estrous cycle. All experimental litters were bred and maintained under specific pathogen-free conditions.

Animals were housed in sterile cages in a room maintained at 24 ± 2 °C with an average humidity of 60 ± 1% and a 12/12-h light:dark cycle and provided access to acidified water and rodent chow ad libitum. After 1 week of acclimation, 21 mice were randomly assigned into three groups (seven per group). Mice in the normal control (NC) group were maintained as described above, whereas mice in both the HTH and probiotics-treatment (PR) groups were regularly transferred into a sterilized climate chamber (model: RXZ-158A, Ningbo Jiangnan Instrument Factory, China) located in the same room. The climate chamber was set as follows: temperature of 30 ± 0.15 °C, humidity of 85–90%, 12 h per day for 8 consecutive weeks. Considering the rapid growth of bacteria especially in the high temperature/humidity condition, food and drinking water were sterilized and renewed each day to avoid contamination.

### Probiotics

Life-space Broad Spectrum Probiotic, purchased from Evolution Health Pty Ltd (VIC, Australia), was used in this study. It is a multi-strain probiotic containing 15 strains of beneficial bacteria and its ingredients can be retrieved from the manufacturer’s website (https://www.lifespaceprobiotics.com/ product/broad-spectrum-probiotic/). From day 50 to 56, animals in the PR group received a single, daily intra-gastric infusion of 50 mg/kg of probiotics suspended in 0.1 mL of saline, whereas mice in the control group were administered an equal volume of saline.

### Fecal collection

Fecal samples were harvested from each mouse on the eighth week, and total DNA was extracted. These were obtained by abdominal pressing at the eighth week and collected into a sterilized tube. Samples were frozen immediately and maintained at −80 °C until processing for bacterial DNA isolation and extraction, which was performed in accordance with previously reported methods^26^. Briefly, the bead beating method was used to collect DNA, which was dissolved with tris-EDTA (TE) buffer after extraction using phenol and chloroform.

### Generation of amplicon library and 16 S rRNA sequencing

Four fecal samples per group were randomly selected for 16 S rRNA sequencing. Gene amplification, cloning, and sequencing of the polymerase chain reaction (PCR) products of bacterial 16 S rRNA were performed at the laboratory of BGI-ShenZhen (Beijing Genomic Institute- Shenzhen Huada Gene Institute, China). PCR amplification of the V5–V4 regions of the bacterial 16 S rRNA gene^27,28^ was performed using universal primers (515 F 5′-GTGCCAGCMGCCGCGGTAA-3′ and 806 R:GGACTACHVGGGTWTCTAAT 907 R 5′-CCGTCAATTCMTTTRAGT-3′), which incorporate unique sample barcode sequences. To generate an amplicon library, 20 ng of each genomic DNA sample, 1.25 U of Taq DNA polymerase, 5 μL of 10 × Ex Taq buffer, 10 mM dNTPs (all reagents from TakaRa Biotechnology Co., Ltd, Dalian, China), and 40 pmol of primer mix were used in a 50-μL reaction mixture. The PCR conditions were as follows: a 5-min initial denaturation at 95 °C; 28 cycles of denaturation at 95 °C (30 s), annealing at 55 °C (30 s), and elongation at 72 °C (45 s), and a final extension at 72 °C for 7 min. PCR products were purified using magnetic beads (Axygen Biosciences, Union City, CA, USA). The concentration of the amplicon library was estimated using the 2100 Bioanalyzer System (Agilent Technologies Inc., Waldbronn, Germany), and equal amounts of amplicons from each sample were pooled together. The sequencing data can be accessed from the European Bioinformatics Institute with accession code PRJEB27835.

### Analysis of sequencing data

Raw data were treated with an in-house pipeline developed based on Mothurv 1.31.2^29^. Primers were removed, low-quality sequences (average quality score of the 30 bp-window < 20) were truncated, and all high-quality reads from individual samples (length > 250 bp) were pooled together. Thereafter, operational taxonomic units (OTUs) were clustered with a 97% identity cut-off using USEARCH (v7.0.1090)^30^. The relative abundance of OTUs was then calculated. OTUs with a relative abundance lower than 0.001% were removed and the distribution of OTUs in every sample was analyzed. Weighted and unweighted UniFrac distance analyses were performed on the basis of OTU abundance and phylogenetic trees. Principal component analysis (PCA) was conducted based on the OTU abundance profile obtained using customized R (3.0.2) scripts. Based on OTU abundances and the taxonomic annotation of OTUs, we obtained relative abundance profiles at the phylum, class, order, family, genus, and species levels^31^. Alpha diversity, beta diversity, and rarefaction curve analyses were performed based on the relative OTU abundance table. The Kruskal-Wallis Test was used to explore the enrichment of bacterial species and functions in different subgroups. The false discovery rate (FDR) was considered to control type I errors and identify more reliable candidates for multiple-group comparisons. In addition, we used PICRUSt to perform functional classification of Kyoto Encyclopedia of Genes and Genomes (KEGG) Orthology (KOs), and Clusters of Orthologs Groups (COGs)^32^.

### Pathological analyses and morphometric quantification

At the end of the study, mice were individually euthanized and their small intestine, caecum, and colon were dissected out, immersed in 10% buffered formalin for 48 h, and processed for hematoxylin and eosin staining, phloxine-tartrazine staining for Paneth cells, and periodic acid–Schiff (PAS)/hematoxylin staining for goblet cells. Light microscopy images under dry lens (20× or 40×) or oil immersion lens (100×) were taken and analyzed with the Leica DMR microscope system (Leica Microsystems, Wetzlar, Germany). Histological evaluation was performed by a pathologist, blinded to the identity of the sample, and the goblet cells per villus were enumerated.

For lipopolysaccharide (LPS) immunostaining, sections were dewaxed and boiled in 10 mM sodium citrate buffer for antigen retrieval, incubated for 1 h in blocking buffer (10 mM Tris-HCl pH 7.4, 0.1 M MgCl2, 0.5% tween-20, 1% BSA, and 5% serum), and incubated with a monoclonal antibody against LPS (Cloud-Clone Corp., Wuhan, China). Staining was observed using a polymer-based visualization system (Gene-Tech Inc, Shanghai, China).

### RNA exaction, reverse transcription, and real-time PCR

Colon and intestine sections were snap-frozen in liquid nitrogen. Tissues were stored at −80 °C and homogenized in liquid nitrogen in accordance with standard procedures for RNA extraction with Trizol reagent (ThermoFisher Scientific, Shanghai, China), as described by the manufacturer. cDNA was prepared by reverse transcription using the PrimeScript RT Master Mix (Takara Biomedical Technology Co., Ltd, China), and quantified via real-time PCR analysis using an ABI 7500 real-time PCR system (Applied Biosystems Inc., CA, USA). Primer sequences were published elsewhere^33^[13] and listed as follows: 5′-GGT GAT CAT CAG ACC CCA GCA TCA GT-3′ (forward) and 5′-AAG AGA CTA AAA CTG AGG AGC AGC-3′ (reverse) for global defensins; 5′-TCA AGA GGC TGC AAA GGA AGA GAA C-3′ (forward) and 5′-TGG TCT CCA TGT TCA GCG ACA GC-3′ (reverse) for *cryptdin-1*; 5′-GCA TGG AAT CTG GGT CAA GAT AAC-3′ (forward) and 5′-AGA AGG AAG AGC AAT CAA GGC TAA G-3′ (reverse) for cryptdin-related sequence (*CRS4C*); *Gapdh* was used as the reference. PCR was performed in triplicate using SYBR Green (Takara Biomedical Technology Co., Ltd, China). cDNA was amplified for 40 cycles using a preset cycling program that included the generation of a melting curve. Thermocycling conditions were as follows: (i) 50 °C for 2 min (activation of AmpErase UNG); (ii) 95 °C for 10 min; (iii) 95 °C for 15 s (denaturation) and 60 °C for 1 min (annealing/extension) for 40 cycles. The ΔΔCT method was used to quantify relative mRNA levels as described in User Bulletin 2 (Applied Biosystems).

### Enzyme linked immunosorbent assay (ELISA)

To quantify the levels of pro-inflammatory cytokines *in situ*, colon sections were snap-frozen in liquid nitrogen immediately and stored at −80 °C until homogenization in tris-HCl buffer containing a protease inhibitor cocktail (Sigma-Aldrich, St. Louis, MO, USA). Mouse TNF-α, IFN-γ, IL-6, and LPS ELISA kits were purchased from Andy Gene Biotechnology Co., LTD (Beijing, China) and used to quantify cytokine levels in the supernatant in accordance with the manufacturer’s instructions. A Varioskan LUX microplate reader (Thermo Fisher, USA) was used for the assay and 4-parameter logistic regression was used for data analysis. Two duplicates per sample were performed and results were averaged.

### Statistical analysis

Results are presented as the mean ± SD values, and multiple-group comparisons were assessed using one-way ANOVA with the LSD multi-comparison test using R software.

### Ethical approval

All experiments were approved by the Animal Care and Welfare Committee of GZUCM (Protocol #2016089) and performed in accordance with the recommendations of the NIH Guide for the Care and Use of Laboratory Animals [National Research Council. Guide for the Care and Use of Laboratory Animals. (2011)].

### Consent for publication

This manuscript has not been published or presented elsewhere in part or in entirety and is not under consideration by another journal. We have read and understood your journal’s policies, and we believe that neither the manuscript nor the study violates any of these.

## Supplementary information

Supplementary File

## Data Availability

The sequencing data can be accessed from the European Bioinformatics Institute with accession code PRJEB27835.
