# Supplementary material for: Gut Dysbiosis with Minimal Enteritis Induced by High Temperature and Humidity
Source: Sci Rep. 2019 Dec 10;9:18686. doi: 10.1038/s41598-019-55337-x (PMC6904617; doi:10.1038/s41598-019-55337-x)
Supplement: Supplementary file 1 — Supplementary File [file 41598_2019_55337_MOESM1_ESM.docx]

Supplementary File

**Title**: Gut Dysbiosis with Minimal Enteritis Induced by High Temperature and Humidity

**Authors**: Song CHEN1, Yuhua ZHENG1, Yiqing Zhou1, Weizhong GUO1, Qin TANG1, Guangli RONG1, Weiwei HU1, Jianbang TANG2*, and Huanhuan LUO3*

**Affiliations:**

^1^Institute of Tropical Medicine, Guangzhou University of Chinese Medicine, No.12 Jichang road, Baiyun district, Guangzhou City, Guangdong Province, People’s Republic of China

^2^Zhongshan Hospital of Chinese Medicine, No.3 Kangxin road, Xi district, Zhongshan City, Guangdong Province, People’s Republic of China

^3^School of Basic Medicine, Guangzhou University of Chinese Medicine, No. 232 Wai Huan Dong Road, University Town, Panyu District, Guangzhou City, Guangdong Province, People’s Republic of China

**Correspondence authors**

Correspondence to Huanhuan LUO : avenluo@gzucm.edu.cn

Correspondence to Jianbang TANG : 85945916@qq.com


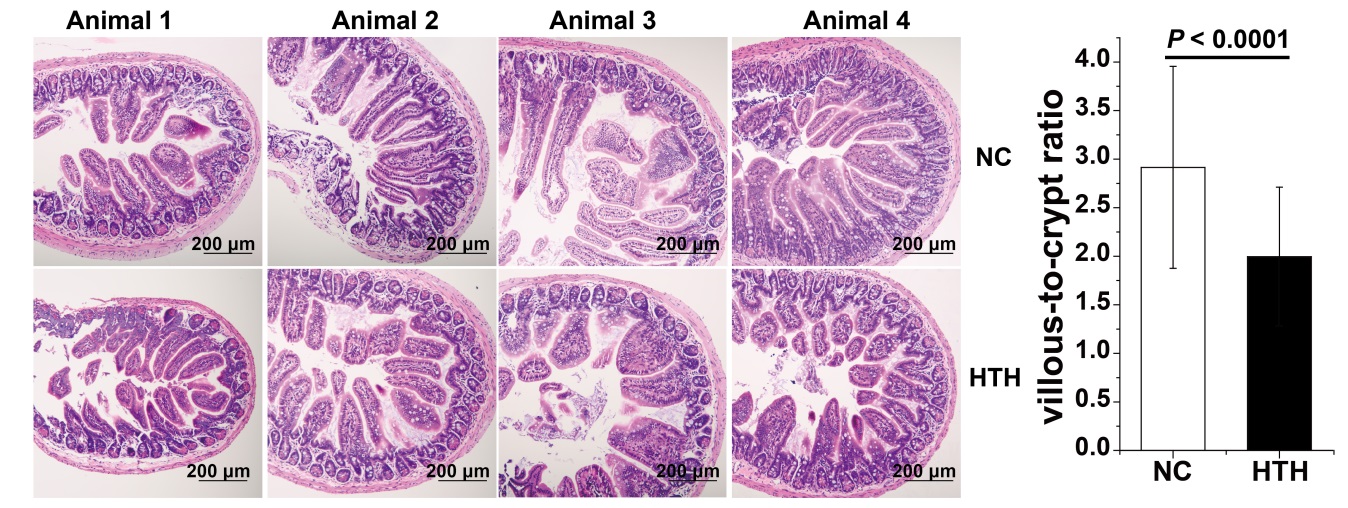


**Supplementary Figure.1. Histomorphology of intestinal tissue in mice.**

The histomorphology of mouse intestine in the NC group (upper) and HTH group (lower) (H&E). The villous-to-crypt length ratios were also showed in columns (*Mean± S.D.*) .


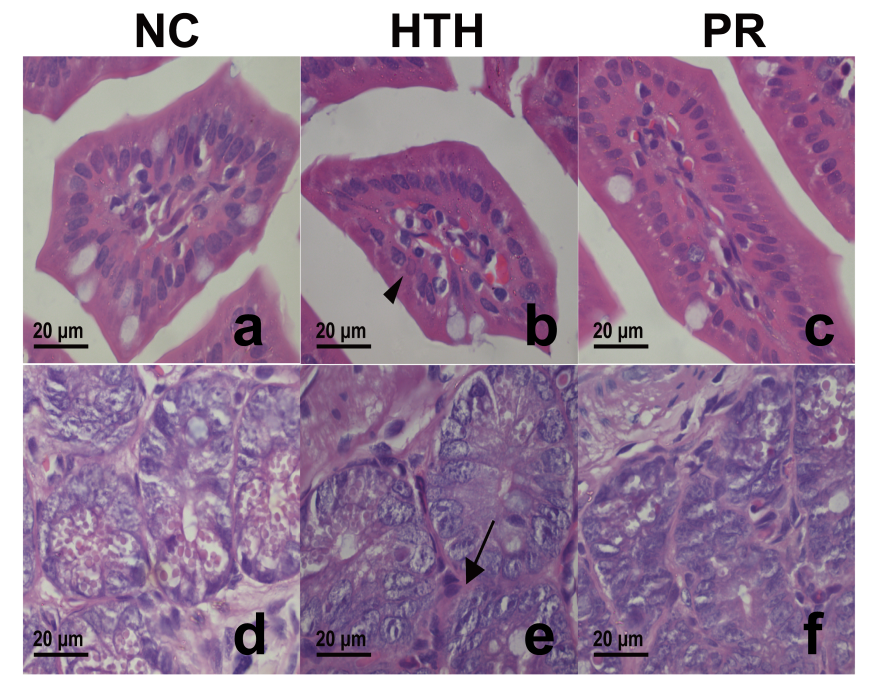


**Supplementary Figure.2. Histomorphology showing minimal inflammation in murine small intestine.**

H&E stained representative images of villi (A, B, and C) and crypts (D, E, and F) of mice in NC group (A, D), HTH group (B, E) and PR group (C, F) were shown. Arrow head indicates the rupture/destruction of the epithelial lining (B), and long arrow indicates the increased capillaries (E & F). Pictures were taken under oil immersion lens, H&E, X1000. NC: normal control group, HTH: high temperature and humidity group, PR: probiotics group. Bar = 20μm
